# Antileishmanial and Antitrypanosomes Drugs for the Current Century

**DOI:** 10.3390/microorganisms12010043

**Published:** 2023-12-26

**Authors:** José María Alunda

**Affiliations:** 1 Department of Animal Health, Faculty of Veterinary Medicine, Universidad Complutense de Madrid, 28040 Madrid, Spain; jmalunda@ucm.es; Tel.: +34-913-943-701; 2 Institute of Industrial Pharmacy, Faculty of Pharmacy, Universidad Complutense de Madrid, 28040 Madrid, Spain

**Keywords:** *Trypanosoma*, *Leishmania*, NTD, HAT, AAT, chemotherapy, One Health, resistance, drug targets, drug discovery

## Abstract

Human infections by trypanosomatids are widely distributed and prevalent in the tropical and subtropical regions. Diseases caused by *Trypanosoma* and *Leishmania* have variable clinical outcomes, ranging from self-healing to fatality, and are considered Neglected Tropical Diseases (NTD). In addition, animal trypanosomiases have a significant impact on animal health and production, apart from their potential role as reservoirs in zoonotic species. Control of these infections is progressing and, in some cases (such as human African trypanomiasis (HAT)), significant reductions have been achieved. In the absence of effective vaccination, chemotherapy is the most used control method. Unfortunately, the therapeutic arsenal is scarce, old, and of variable efficacy, and reports of resistance to most antiparasitic agents have been published. New drugs, formulations, or combinations are needed to successfully limit the spread and severity of these diseases within a One Health framework. In this Special Issue, contributions regarding the identification and validation of drug targets, underlying mechanisms of action and resistance, and potential new molecules are presented. These research contributions are complemented by an update revision of the current chemotherapy against African *Trypanosoma* species, and a critical review of the shortcomings of the prevailing model of drug discovery and development.

## 1. Diseases by Trypanosomatids: World Perspective

Trypanosomiases and leishmaniases are globally distributed vector-borne parasitic infections that affect humans, domestic animals, and wildlife. They are considered Neglected Tropical Diseases (NTDs), although their current geographical distribution extends beyond tropical areas [1]. The importance of these infections was recognized long before the actual aetiological agents causing them were identified. Thus, several diseases were described in humans (e.g., kala-azar (*Leishmania donovani* and *L. infantum*), chiclero’s ulcer, uta (*L. mexicana*, *L.* (*Viannia*) *braziliensis*), sleeping sickness (*Trypanosoma brucei gambiense*, *T. b. rhodesiense*), and Chagas’ disease (*T. cruzi*)). Similarly, diseases caused by trypanosomatids were also described in animals, (e.g., nagana (*T.b. brucei*, *T. vivax*, *T. congolense*), mal de caderas (*T. equinum*), and dourine (*T. equiperdum*)). Not surprisingly, some of these infections were among the first tropical diseases to be addressed by scientists, medical doctors, and veterinarians.

Despite the economic and social interest in controlling these infections, the results obtained have generally fallen short of expectations. Several reasons can be identified, including the complexity of controlling diseases with vector species with different ecological and ethological characteristics; the diversity of primary vertebrate hosts and the abundance of reservoirs; the limited success of environmental controls; their chronic course (which implies the presence of potential carriers); the variation in parasite pathogenicity and immunogenicity; and the lack of a complete understanding of the elicited immune responses in target species. No vaccine against human infections with *Trypanosoma* or *Leishmania* is available, and the marketed vaccines against zoonotic *L. infantum* in dogs have serious drawbacks.

## 2. Chemotherapy as a Major Actor in the Control of Trypanosomatids

Products are more readily accepted than procedures. Although it is considered that long-term strategies to control these NTDs should rely on a range of activities (e.g., education of human target populations, sanitation to limit vectors, adequate housing, and on-site medical and veterinary services, among others), it is clear that accessible and affordable antiparasitic drugs, with a tolerable efficacy–toxicity balance, easy administration, and rapid onset of action, are relevant tools to control human parasitic infections and to also improve the health and productivity of animals and, ideally, to reduce the risk of transmission of zoonotic parasites in a One Health context.

### 2.1. Chemotherapy of Human African Trypanosomiases (HAT)

Historically, Human African Trypanosomiases (HATs) were the first trypanosomiases over which control was attempted [2]. Since Fowler’s solution (1% potassium arsenite) in 1858, arsenic preparations and formulations have been employed for the treatment of sleeping sickness. Atoxyl (aminophenylarsenic acid) was, despite its name, too toxic to be used, but tryparsamide (1919, Walter Jacobs & Michael Heidelberg) was the first drug to treat the second stage of sleeping sickness, and remained the drug of choice for human and animal trypanosomiases until the 1960s. Melarsoprol, a less toxic derivative of melarsen oxide, was introduced in 1949. Despite the limitations, it is still the only effective drug for chemotherapy of the second stage of *T. b. rhodesiense* sleeping sickness [2]. Bayer synthesized the first useful treatment for HAT (Bayer 205), which was eventually marketed under the name of Germanin [3]. This compound was followed by several drugs from different pharmaceutical companies and used in different regions. As a result, HAT was under control by the mid-1960s, with fewer than 5000 cases reported in the African continent [4].

Reduced vector control due to the banning of DDT spraying in the late 1970s, together with reduced disease prevention, led to a steady increase in HAT [3] (among other NTDs) reaching levels close to those found before WW2 by the end the 1990s. Efforts by the WHO, international cooperation, national control programs, Non-Governmental Organizations (NGOs), and new treatments regimes and products (e.g., Eflornithine, Nifurtimox, Fexinidazole, and combination therapy) have reduced the burden of HAT. Although the information is very variable, a reduction of HAT from over 6500 cases/year in 2011 and 2184 in 2016 to 663 in 2020 has been reported (https://www.statista.com/statistics/871510/human-african-trypanosomiasis-cases-reported-and-expected-worldwide/; https://unitingtocombatntds.org/reports/5th-report/) (accessed on 28 November 2023).

This success has been achieved with a reduced therapeutic arsenal: Pentamidine, Suramin, Melarsoprol, Eflornithine, Fexinidazoe, and the combination of DFMO + Nifurtimox [5]. Pentamidine and Suramin have historically been the drugs of choice for the treatment of blood stage *T. b. gambiense*-HAT and *T. b. rhodesiense* HAT, respectively. Due to the encephalopathies associated with the use of melarsoprol in the second stage of the disease (central nervous system involvement), this drug is not recommended for *T. b. gambiense* HAT, and a combination therapy of eflornithine and nifurtimox is used instead [6]. Despite this success, in terms of the drugs employed to fight HAT, only Eflornithine is a new chemical entity (NCE), and was registered in 1990. Resistance has also been reported [7,8,9].

### 2.2. Treatment of Animal Trypanosomiasis

Animal trypanosomiases affect all domestic species (sheep, goats, cattle, equines, pigs, camelids), and are present in Africa, South America, and Asia; the infection is also present in wild animals [10,11]. The zoonotic potential of animal trypanosomiases is generally low, although the reservoir role of some domestic animals has been reported [12], and the main impact of the disease is economic. African animal trypanosomiasis (AAT) constrain agricultural production (>50% reduction of milk and meat) in areas of Africa with the greatest potential for expanded agricultural production in the continent [13]. The reduced income of farmers in endemic areas due to animal mortality, reduced production, and the cost of trypanocidal drugs, dampening the improvement of their life conditions and investments. Furthermore, in a One Health scenario, control of HAT transmission will affect the AAT transmission, and vice versa [8,14,15]. AAT is mainly treated with homidium, isometamidium, quinapyramine, diminazene aceturate, and melarsomine [5]. As with other animal diseases, effective control of AAT requires not only the availability of better and more affordable trypanocidal drugs (a need that remains unmet), but also improved management systems that consider the drivers and behavioral practices of farmers, as well as the control of treatment failures, to be incorporated into sustainable solutions [16,17].

### 2.3. Treatment of Chagas Disease

Chagas disease, caused by *Trypanosoma cruzi* remains a major social and public health problem in South America. The parasite is usually transmitted by blood-sucking bugs (Triatominae, for example *Triatoma infestans* or *Rhodnius prolyxus*). The parasites enter the human body through small wounds in the skin that are contaminated with the feces of the vectors. The vectorial transmission occurs in South, Central, and North America, including Mexico and the southern states of the USA [18]. Human pressure and plasticity of *T. cruzi* have resulted in non-vectorial transmission through healthy mucosa, solid organ transplants, and blood transfusions. Thus, *T. cruzi* infection is also present in Europe, Canada, Australia, and Japan, through human migration [19].

Nifurtimox and benznidazole are the only two effective treatments for *T. cruzi* infections [20,21]. However, both prodrugs are only effective in the acute or early infection phases, and they can cause adverse effects during their use. Resistance or treatment failures have been reported in early phases and gradient activity (0–100%), depending on the isolate/strain [19,20,21].

### 2.4. Chemotherapeutic Control of Leishmaniasis

Leishmaniasis is a vectorial parasitic disease caused by several species of the genus *Leishmania* (Trypanosomatidae). Human infection is present in almost 100 countries, although ca. 90% of the cases are reported from 15 countries in Africa, South and Central America, and Asia [1,22]. Depending on the aetiological agent involved, and on the immune status of the infected individual, several clinical presentations have been described: cutaneous (CL), mucocutaneous (MC), and visceral leishmaniasis (VL), as well as post-kala-azar dermal leishmaniasis (PKDL) [1]; VL caused by *L. donovani* and *L. infantum* is the second most lethal parasitic disease affecting humans [23]. The various forms of CL are, by far, the most frequent presentation of the disease, and are caused by *Leishmania* species in the Americas (e.g., *L. braziliensis*, *L. panamensis*, *L. peruviana*, *L. amazonensis*, *L.mexicana*), Eurasia, and Africa (*L. aethiopica*, *L. major*, *L. tropica*). With the sole exception of *L. donovani* and *L.tropica*, these species are zoonotic [24], especially *L.infantum,* with dogs being the main reservoir of human infections [25].

Current chemotherapy of leishmaniasis includes miltefosine, pentavalent antimonials (Sb^V^), amphotericin B, paromomycin, pentamidine, azoles and allopurinol [26,27,28,29]. Approval of miltefosine for human use was a breakthrough, since it can be orally administered, thus facilitating patients’ compliance [30]. Efficacy of the compounds is variable, depending on the *Leishmania* species and geographical areas, and most of them present significant side effects, including teratogenicity. Development of liposomal formulations of amphotericin B (e.g., Ambisome^®^, Abelcet^®^) improved drug efficacy by targeting the drug to the sites of infection [31,32]. Unfortunately, the less toxic and more efficacious formulations are hardly affordable by patients living in the endemic areas. Moreover, emergence of resistance to the first-line treatments (e.g., antimonials) and to the most recently marketed drug, miltefosine, has been reported [27]. Under such circumstances, there is an urgent need for new drugs against leishmaniasis.

### 2.5. Chemotherapy of Trypanosomiases and Leishmaniasis: Challenging Future and Prospects

Parasites and hosts are constantly evolving to adapt to new conditions. Under these conditions, any alteration will have repercussions on the unstable equilibrium of their populations. Domestication and the advent of agriculture brought about profound changes in parasite–host relationships, including the selection of exploitable species, parasites, and biological cycles compatible with established livestock systems; zoonotic cycles; and the increased density of a few domesticated species. Although “remedies” were used for some parasitic diseases, it was the birth of chemotherapy that was the turning point in bringing hosts and, more importantly, parasites into contact with synthetic chemicals that had not previously existed on Earth. The contact of these xenobiotics with the parasitic agents created a new landscape in which new evolutionary forces determined the selection of parasite variants capable of resisting the lethal action of the synthetic compounds. Moreover, inadequate dosing (especially in livestock) increased the selection pressure. As a result, the need for antiparasitic drugs and the growing resistance to chemotherapy posed a Scylla-and-Charybdis dilemma for animal production and human health.

The therapeutic arsenal to treat trypanosomatid infections is reduced, and resistance to the first-line drugs has been reported in *Trypanosoma* and *Leishmania* (see above). This scenario has stimulated research efforts to identify new drug targets in HAT-causing *Trypanosoma* (benzoxaboroles, pafuramidine (DB289)) [33]; *T. cruzi* (e.g., ubiquitin-proteasome, cruzipain, Fe-SOD and TR, purine salvage, trans-sialidase, or ergosterol biosynthesis) [34], and *Leishmania* [35]. The availability of powerful research tools could help to identify and validate the essential genes and, hence, new chemotherapeutic targets [36]. Alternatively, the repurposing, combination, and new presentations of “old drugs” (e.g., melarsoprol–cyclodextrin complexes) could reduce treatment failures and the emergence of resistance [21,33].

## 3. The Special Issue on “Chemotherapy of *Leishmania* and *Trypanosoma* Infections: Lost in Translation?”

Global efforts to control the spread and severity of diseases caused by Trypanosomatids in humans, and improve living conditions in NTD-affected areas, have achieved remarkable results. The beneficial effects of mass treatments of humans, and improved access to health services, water sanitization, and better nutrition are clear, and the reduction in human cases of “sleeping sickness” (HAT) is one example. However, similar success has not been achieved against CL and disfiguring MC, nor in controlling *T. cruzi*. For AAT, in addition to their potential zoonotic role, effective control of nagana by *T. brucei* is yet to come. Resistance, unaffordability, low efficacy, long treatment courses, and adverse side effects characterize some of the available drugs. The continued efforts of scientists are therefore still needed. This Special Issue of *Microorganisms* aims to contribute to the improvement of chemotherapeutic control of *Trypanosoma* and *Leishmania* infections in both humans and domestic animals. The contributions address some of the most relevant issues: identification of new drug targets; resistance mechanisms and impacts; and the potential value of natural and natural-derived molecules. In addition, an update of the current chemotherapy of HAT and ATT, and a draft program to improve the efficiency of the process for developing new drugs or combinations against *Trypanosoma* and *Leishmania* infections, are included.

NCEs against trypanosomatids have been scarce, and natural or natural-derived molecules could be a suitable alternative. In a noteworthy contribution to this Special Issue, the antileishmanial activity of betulin and its derivatives against promastigotes and intracellular amastigotes, and the underlying mechanism of action (MoA), were investigated. Two molecules showed high potency and selectivity against the intramacrophage stage of *L. amazonensis*. These properties, together with the induction of superoxide production by macrophages by one of the synthesized compounds, support the further exploration of this series to develop new chemotherapeutic agents against leishmaniasis [37]. Ideally, the identification of the therapeutic target and MoA of currently used or new drugs would provide a rational background for further improving treatments and preventing the emergence of resistance. Working with HAT-causing *T. brucei*, and using RNA interference (RNAi) libraries a series of C7-substitud nucleoside analogues, an important contribution to this Special Issue has evidenced the involvement of adenosine kinase and 4E interacting protein into the MoA of antitrypanosomal nucleoside analogues. Furthermore, the essential nature of the protein for parasite growth and infectivity suggests the identification of a potential new drug target [38]. GDP-mannose pyrophosphorylase (GDP-MP), an enzyme involved in the mannosylation pathway, is considered an attractive therapeutic target for the development of antileishmanial drugs. Another contribution to this topic investigated the impact of single mutations (aspartate instead of alanine, position 258) in *L. infantum* GDP-MP (LiGDP-MP) on substrate specificity, MoA, and kinetic parameters. No substantial modifications were found when compared to the homolog molecule from anthroponotic *L. donovani* (LdGDP-MP). Therefore, the potential advantage of modifying residue 258 for *L. infantum* should be investigated [39]. The mechanistic explanation of the resistance phenomena is strongly linked to the MoA, but our knowledge is largely incomplete. A relevant contribution to this Special Issue [40] presents an investigation of the effect of an inhibitor of HMG-CoA reductase (simvastin) of the sterol biosynthesis pathway in drug-resistant *L. amazonensis*. The results obtained have shown that simvastatin-resistant *L. amazonensis* parasites (LaSimR) underwent reprogramming of the sterol metabolism, and showed cross resistance to ketoconazole (a C14DM inhibitor), miltefosine, tunicamycin, and several protease inhibitors. The regulation of sterol biosynthesis in simvastin-resistant *Leishmania*, and the cross resistance found with both sterol-related and unrelated drugs, support the further investigation of this route. The aminoglycoside antibiotic aminosidine (paromomycin, PMM) is a repurposed treatment of leishmaniasis (VL, CL) with an initial efficacy comparable to that of antimonials. Its use has been hampered by the rapid emergence of resistant strains of *Leishmania*. MoA in bacteria involves the inhibition of protein synthesis by interacting with the ribosomal subunits. However, no causal molecular mechanisms for *Leishmania* PMM resistance have been identified so far. A relevant contribution in this Special Issue [41] addresses this aspect by investigating genomic variations found in twelve experimentally selected *L. donovani* clonal lines resistant to PMM compared to sensitive lines. Whole genome sequencing allowed identification of eleven short nucleotide variations and copy number alterations in 39 genes correlated to PMM resistance. The identified genes were involved in transcription, translation, protein turn-over, virulence, mitochondrial function, signaling, and vesicular trafficking. The multiple actions of PMM suggest a multifactorial origin of resistance of *Leishmania* to PMM.

An excellent update of the available medications against HAT and AAT, considering the pharmacological properties of the therapeutic options against *T. brucei* infections, is included in this Special Issue [42]. In particular, this review highlights the relevance of the One Health approach, the reservation of several antileishmanial drugs for human use, the need for clearer and more defined guidelines in the use of drugs designed for HAT and AAT, and monitoring to avoid inter-species cross-resistance and to maintain the therapeutic activity of the drugs. Finally, this Special Issue includes a critical review of the need to rethink the prevailing model of drug discovery against trypanosomatid infections and its low efficiency. This contribution supports the need to improve the drug discovery and development process by establishing common goals in collaborative research, especially in private–public consortia, the role of pharmacological properties in the initial selection of molecules, the use of adequate surrogate models with high predictive value, the complete study of experimental animals in the in vivo trials, and reconsideration of the value of Target Product Profile (TPP) guidelines [43].
